# Inertial Migration of Neutrally Buoyant Spherical Particles in Square Channels at Moderate and High Reynolds Numbers

**DOI:** 10.3390/mi12020198

**Published:** 2021-02-14

**Authors:** Yanfeng Gao, Pascale Magaud, Lucien Baldas, Yanping Wang

**Affiliations:** 1 College of Engineering and Applied Sciences, State Key Laboratory of Analytical Chemistry for Life Science, Nanjing University, Nanjing 210023, China; gaoyanfeng@nju.edu.cn; 2 Institut Clément Ader, Université de Toulouse, CNRS, INSA, ISAE-SUPAERO, Mines-Albi, Université de Toulouse, 3 rue Caroline Aigle, 31400 Toulouse, France; pascale.magaud@insa-toulouse.fr; 3 Sino-French Engineer School, Nanjing University of Science and Technology, Nanjing 210094, China; yanping.wang@njust.edu.cn

**Keywords:** microfluidics, inertial migration, high Reynolds numbers, particle ordering

## Abstract

The inertial migration of particles in microchannel flows has been deeply investigated in the last two decades. In spite of numerous reports on the inertial focusing patterns in a square channel, the particle inertial focusing and longitudinal ordering processes remain unclear at high Reynolds numbers (>200) in square microchannels smaller than 100 µm in width. Thus, in this work, in situ visualization of particles flowing in square micro-channels at Reynolds numbers *Re* ranging from 5 to 280 has been conducted and their migration behaviors have been analyzed. The obtained results confirm that new equilibrium positions appear above a critical *Re* depending on the particle to channel size ratio and the particle volume fraction. It is also shown that, for a given channel length, an optimal Reynolds number can be identified, for which the ratio of particles located on equilibrium positions is maximal. Moreover, the longitudinal ordering process, i.e., the formation of trains of particles on equilibrium positions and the characterization of their length, has also been analyzed for the different flow conditions investigated in this study.

## 1. Introduction

Particle sorting, separation, and manipulation in a suspension are essential steps for a wide range of applications in the chemical industry, pharmaceutical preparation, clinical diagnostics, etc. To address these needs, various particle manipulation technologies have been developed in recent years. The related methods can be mainly classified into two types, according to the nature of the forces involved, i.e., active methods using external force fields and passive methods only based on internally induced forces. For the active methods, forces of different nature can be used, like electrical forces [1], acoustic radiation forces [2], magnetic forces [3], or even optical forces [4,5]. In contrast, passive methods such as hydrophoresis [6], deterministic lateral displacement [7,8], and inertial focusing [9] mainly take advantage of hydrodynamic forces. Among the above methods, the inertial focusing process has the advantage to be robust, simple, and continuous. Thus, it shows a great potential for the development of low-cost and high-throughput microsystems for various applications, such as separating, concentrating, counting, detecting, sorting, or mixing of particles in suspensions [10,11].

The inertial migration of neutrally buoyant particles was first quantitatively high-lighted by Segre and Silberberg in the 1960s [12]. Millimetric particles, flowing in a millimeter-scale straight cylindrical tube, were observed to migrate at moderate Reynolds numbers across the streamlines and to focus on an annulus with a radius of ~0.6 times the tube radius. During the course of investigating this “tubular pinch effect” phenomenon, the authors also noticed the phenomenon of particle alignment. A few decades later, thanks to the advances in microfabrication techniques, similar phenomena were also observed in microscale channels.

Di Carlo et al. [13] first observed that neutrally buoyant particles flowing at moderate Reynolds numbers focused only on four equilibrium positions located near each channel wall along their symmetry planes. These results were confirmed by numerical simulations [11,14]. However, it was also reported that the number and locations of equilibrium positions could vary depending on the flow conditions. Abbas et al. [14] highlighted that, in square microchannels, particles concentrate in the channel centerline at low flow inertia (typically Reynolds number *Re* < 1). Miura et al. [15] experimentally observed the presence of eight equilibrium positions simultaneously located at the centers of the channel faces and at the corners of the cross-section in square millimetric channels for Reynolds numbers higher than 250. More recently, Shichi et al. [16] experimentally investigated the inertial migration of neutrally buoyant spherical particles (from 30 to 70 µm in diameter) suspended in channel flows of sub-millimetric square cross sections (from 400 to 800 µm) in the range of Reynolds numbers from 1 to 800. They confirmed that, at *Re* smaller than a low critical value *Re_c1_* (~100), particles are focused at four equilibrium positions, located at the centers of channel faces. This was called the first regime of migration. At *Re* larger than a higher critical value *Re_c2_* (~450), four additional equilibrium positions are observed near the channel corners. This was called the third regime of migration. At intermediate *Re*, between the previously mentioned two critical values, eight focusing positions of particles are observed located on a heteroclinic orbit joining the channel face and corner equilibrium positions and called the second regime of migration. As *Re* increases above *Re_c1_*, the azimuthal angle of the intermediate equilibrium positions increases up to π/4 where they merge with the corner equilibrium positions at *Re* = *Re_c2_*.

Nakagawa et al. [17] showed numerically that the inertial migration to eight equilibrium positions still takes place in a two-stage process. In the first stage, particles move toward the annulus and, in the second stage, they travel along the annulus toward equilibrium positions. According to them, both face-centered and corner equilibrium positions are stable in this regime of migration. Thus, the corner equilibrium positions exhibit a transition between unstable (during the first regime of migration) and stable states (in the third regime of migration). The reason why the corner equilibrium positions exhibit this transition from unstable to stable states is still unknown. Shear-induced and wall-induced lift forces act predominately during the first stage of migration, while rotation-induced lift force become predominant in the second stage of migration where the two former forces balance each other. This transition might, thus, be linked to an evolution of the rotational-induced lift force. Nakagawa et al. [6] suggested that the presence of two opposite rotational-induced lift forces during the second stage of migration directed toward the channel face centers for particles located in the equilibrium annulus near the face centers and directed toward the channel corners for particles located in proximity to the corners, might be due to the inter-particle interaction.

Pan et al. [18] and Yuan et al. [19] investigated, experimentally and numerically, the flow field around a single focused particle in a confined microchannel. They concluded that a focused particle in a confined flow modifies the velocity distribution and induces a secondary flow in its vicinity. As *Re* increased, the velocity distribution becomes different enough from that of a Poiseuille flow to significantly affect the inertial migration of the particle. This modification in the flow field might also be involved in the modification of the migration regime.

Regarding the longitudinal ordering phenomenon, Matas et al. [20] first conducted an in-depth investigation of particle self-assembly in circular pipes and suggested that the “reversing streamlines” are essential for the formation of particle trains. After that, a similar particle alignment phenomenon was observed in square [13] and rectangular microchannels [21]. Lee et al. [22] suggested a mechanism for the dynamic self-assembly process, based on a model of interparticle force between a slow leading particle and a fast lagging one. Pan et al. [18] experimentally investigated with a micro-PIV system the flow structures induced by in-line particle trains in a rectangular microchannel at moderate *Re* in order to better understand the mechanisms of interaction among particles, fluid, and channel walls. Gao et al. [23] statistically investigated the dynamics of particle ordering in square microchannels and the influence of the particle to channel size ratio, Reynolds number, and particle concentration on the formation of particle trains. They also explored the particle ordering phenomenon in a bi-disperse suspension, illustrating the formation of sequential mixed trains [24]. 

Some of the previously cited works have considerably enhanced the knowledge about lateral migration of particles at high Reynolds numbers but in channels with dimensions of several hundred micrometers that are too large for applications in typical lab-on-a-chip devices (usually smaller than 100 µm). Besides, the longitudinal ordering of particles observed by several authors [19,20,21,22,23,24] at moderate *Re* was rarely reported at high Reynolds numbers.

To shed light on these phenomena at high *Re* in microchannels with dimensions smaller than 100 µm, we experimentally studied the transport of neutrally buoyant spherical particles in square microchannel flows at Reynolds numbers ranging from 5 to 280. First, the particle distributions obtained at different Reynolds numbers were investigated and compared with the literature with a particular emphasis on the high Reynolds regime. Second, the influence of the particle to channel size ratio and the particle volume fraction (volume occupied by the particles over the total volume of the suspension) on the lateral migration behavior was analyzed. Finally, the evolution of the number of particles in train and of the composition of the trains with *Re* has been highlighted.

## 2. Materials and Methods

**Figure 1:**
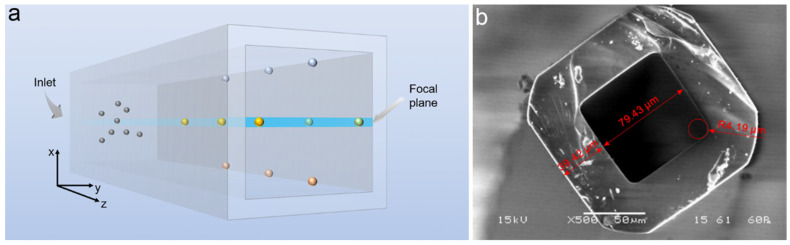
Scheme of the experimental setup. (**a**) Schematical illustration of particles flowing in the microchannel. (**b**) Scanning electron microscopy (SEM) image of the microchannel’s cross-section.

The spherical particles used for this study are made of polystyrene (Microparticles GmbH^®^, Berlin, Germany) with diameters *d_p_* = 5.3, 8.44, and 15 µm and a density *ρ* = 1050 kg m^−3^. The fluid density was matched to the particle one by using a mixture of 23% glycerol and 77% deionized water. The particle volume fraction *Φ* varied from 0.02% to 1%. The suspension flowed in square borosilicate micro-channels (Vitrocom) with 80 × 80 μm^2^ inner section and 10-cm and 30-cm lengths (see Figure 1). The flow rates generated by a syringe pump (PDH 4400, Harward Apparatus, Holliston, MA, USA), ranged from 667 nL/s to 33 µL/s corresponding to *Re* from 5.6 to 280 (*Re* = *UH*/*ν* where *U* is the flow mean velocity, *H* is the channel height, and *ν* is the kinematic viscosity of the fluid). Particle positions in the channel were observed in situ thanks to an Olympus microscope (Tokyo, Japan) (B× 51) and images were recorded at several positions along the channel length by a high-speed camera (Photron Fastcam SA3, Tokyo, Japan) with a focal plane located at the midplane of the channel. Particles with clear outlines were recognized as in-focus particles (i.e., within the focal plane), while others with blurry outlines were regarded as out-of-focus particles. This strategy allowed a rough reconstruction of a 3D particle distribution in the microchannel.

**Figure 2:**
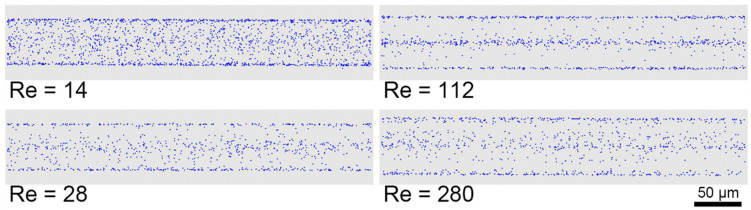
Overall particle distributions observed from the channel midplane for different *Re* at a distance *z*/*H* = 1000 from the channel inlet and for particles of diameter *d_p_* = 8.44 µm and a particle volume fraction *Φ* = 0.05%.

For each experimental condition, at least 2000 images were captured to yield a satisfactory result in terms of statistical analysis. The localization of in-focus and out-focus particles on each image was identified by data processing using an in-house MATLAB code including the following steps: channel walls detection, background noise removing, binarization, and particle identification. The positions of particle centers obtained from the 2000 recorded images were then collected on one reference image to visually show the distribution of particles in the channel, as illustrated in Figure 2. A statistical analysis was then performed to obtain a particle distribution function. Both the experimental setup (as shown in Figure 1) and the image processing method have been described in detail in previous works [14,23,24].

## 3. Results

### 3.1. Lateral Migration Process at Moderate and High Reynolds Numbers

Figure 2 displays the overall particle distributions in the midplane of the channel at *Re* = 14, 28, 112, and 280, observed at *z*/*H* = 1000. These distributions were obtained by superposing in the same image the centers of particles identified in the 2000 acquired images. At *Re* = 14, particles are randomly distributed over the image, indicating a relatively uniform distribution in the cross-section. As the Reynolds number increases from 14 to 112, particles were seen to concentrate in lines located near the front and back walls and on the centerline of the channel: particles are migrating toward the four face-centered equilibrium positions. At *Re* = 112, the focalization seems to be nearly fully developed. Since these images have been taken at a fixed distance from the channel inlet (*z*/*H* = 1000), total migration was not completed at *Re* < 112. At *Re* = 280, the particles were no longer concentrated in lines, but were distributed more widely in the channel cross-section, confirming that the well-known migration regime at four spots located near the centers of the channel faces at moderate Reynolds numbers is no more valid for *Re* = 280.

**Figure 3:**
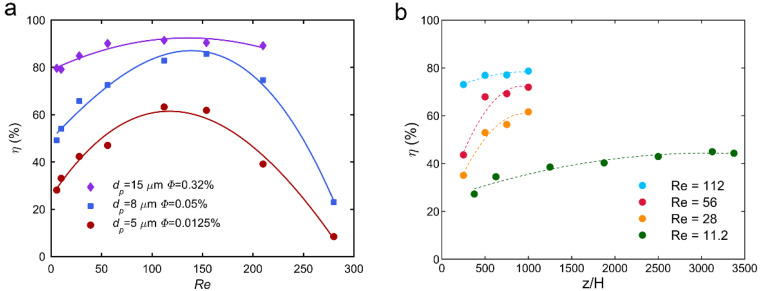
(**a**) Focusing degree *η* at *z*/*H* = 1000 from channel inlet and *Φ* = 0.05% with respect to *Re*, for a different particle to channel size ratios *d_p_*/*H*. (**b**) Focusing degree *η* for a particle to channel size ratio *d_p_*/*H* = 0.11 (*d_p_* = 8.44 µm) and *Φ* = 0.05% with respect to the dimensionless distance from the channel inlet *z*/*H* for different Reynolds numbers.

A parameter called “focusing degree” (denoted as *η*) is introduced to quantify the development of the face-centered focalization. It is defined as the ratio of particles focused at the four face-centered equilibrium positions over the total number of particles in the microchannel [23]. Figure 3a illustrates the focusing degree *η* for various *Re* at a distance from the channel inlet *z*/*H* = 1000. For *d_p_*/*H* = 0.11 (particles of diameter *d_p_* = 8.44 µm), *η* increases with increasing *Re*, reaches a maximum at a critical Reynolds number *Re_c_* ~ 120 in the present conditions, and then decreases. This confirms that, at a fixed distance from the channel inlet, the focalization is first enhanced by increasing the Reynolds number up to a maximum where the focalization becomes fully-developed. For *Re* > *Re_c_*, *η* decreases, indicating that the particles are no longer concentrated on the four face-centered equilibrium positions.

Figure 3b shows the focusing degree of 8.44-µm particles flowing at *Re* = 11.2, 28, 56, and 112 at normalized distances *z*/*H* from the channel inlet ranging from 375 to 3375. The focusing degree first increases acutely with an increasing measurement position, indicating that the lateral migration progresses along the channel length, before reaching a constant value corresponding to the fully developed focalization state. It can also be seen that, for Reynolds numbers lower than 112, the fully developed focusing degree increases and the distance needed to reach the fully developed focalization state decreases with increasing *Re*.

**Figure 4:**
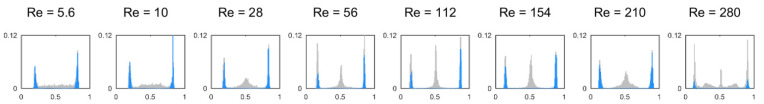
Spatial distributions (Probability Density Functions, PDFs) of 8.44-µm particles in the channel midplane over normalized lateral positions for various *Re* at a fixed distance from the channel inlet *z*/*H* = 1000 and a particle volume fraction *Φ* = 0.05%. Spatial distribution of all the particles identified in the acquired images are represented in grey, while the blue PDFs correspond to particles located in the midplane.

The pictures of the particle distributions presented in Figure 2 are statistically analyzed and redrawn in Figure 4. Particles in the midplane (identified by their specific appearance in the focal plane as detailed in Reference [23]) are colored in blue, while the distribution of all the particles (in and out of the focal plane) is represented in grey. For *Re* = 10, the two blue peaks observed near the walls and the uniform distribution of particles in grey indicate that the particles have migrated toward the channel walls and form a square annulus. Abbas at al. [14] showed that the face-centered focalization at moderate *Re* takes place in a two-stage process: a first stage of lateral migration where particles move laterally toward an annulus (also called a ring) located near the perimeter of the cross-section, which is followed by a second stage of cross-lateral migration where they travel along the annulus toward the face-centered equilibrium positions. At *Re* = 10, the first stage of migration seems fully developed. At *Re* = 112, the particles are accumulated near the centers of the channel walls, indicating that the cross-lateral migration is finished. At *Re* = 154 and 210, the peaks are wider. Particles are more widely distributed and the four face-centered spots are no longer the only equilibrium positions. At *Re* = 280, four new peaks arise in the distribution corresponding to new equilibrium positions.

These distributions and the location of the peaks at *Re* = 280 corresponding to other equilibrium positions are consistent with the experimental and numerical results of Shichi et al. [16] obtained at *Re* = 280.

From *Re* = 112 to *Re* = 280, a transition of focusing positions from face-centered spots toward the corner area can be easily observed in Figure 4. The underlying mechanism for explaining this transition is still unclear, but it can be related to the flow field change at high *Re*, as suggested by Pan et al. [18] and Yuan et al. [19].

### 3.2. Effect of Particle Size on the Lateral Migration Process

The evolution of the focusing degree with *Re* is presented in Figure 3a for three particle to channel size ratios: *d_p_*/*H* = 0.11 for particles of diameter *d_p_* = 8.44 µm, *d_p_*/*H* = 0.066 for particles of diameter *d_p_* = 5 µm, and *d_p_*/*H* = 0.19 for particles of diameter *d_p_* = 15 µm. Three different particle volume fractions *Φ* were chosen for the different particle sizes in order to ensure the same number of particles in a given volume. The evolutions are similar for the three suspensions: the focusing degree first increases with increasing *Re*, reaches a maximum for a given critical Reynolds number *Re_c_*_1_, and then decreases. This confirms that, at a fixed distance from the channel inlet, the focalization is first enhanced by increasing the Reynolds number up to a maximum where the focalization becomes fully developed. The decrease of *η* observed for *Re* higher than *Re_c_*_1_ is due to a switch in the migration regime and, thus, the emergence of other equilibrium positions, as explained in the previous section. However, increasing the particle to channel size ratio *d_p_*/*H* increases the maximum focusing degree *η*. In other words, larger particles are more concentrated on the four face-centered equilibrium positions than smaller ones. This can be explained by the fact that larger particles experience larger inertial lift forces, as highlighted in the work of Di Carlo et al. [11] who found an inertial scaling for the shear-induced lift force involving the particle diameter at the power of 3. Thus, at the same observing position from the channel inlet, larger particles migrate faster than smaller ones, which leads to a higher focusing degree.

Furthermore, it can be observed that the shapes of the three curves are very different. Small particles have a focusing degree curve rather sharp whereas large particles present a focusing degree curve to be rather flat. In other words, at a given distance from the channel inlet, the range of Reynolds numbers where most of the small particles are focused is small. They need a high Reynolds number to reach the fully-developed focalization and they rapidly leave the face-centered equilibrium positions toward new equilibrium positions when *Re* increases. The range of Reynolds numbers where most of the larger particles are focused at the four face-centered equilibrium positions is, in contrast, very large. At a given distance from the channel inlet, most of the large particles reach their equilibrium position even for low *Re*, indicating that they suffer larger shear-induced lift force and they stay on these four face-centered equilibrium positions for larger Reynolds numbers before to move toward new equilibrium positions. In other words, the critical Reynolds number *Re_c_*_1_ at which the intermediate equilibrium positions emerge, increases with increasing *d_p_*/*H*. This finding is consistent with the results of Shichi et al. [16]. They showed that the values of *Re_c_*_1_ and *Re_c_*_2_ depend considerably on the size ratio: an increase in *d_p_*/*H* from 0.075 to 0.175 increases *Re_c_*_1_ whereas it decreases *Re_c_*_2_, i.e., the *Re* value at which the corner equilibrium positions appear. They showed that the range of *Re* for the second regime where particles are focalized on the intermediate equilibrium positions varies significantly depending on *d_p_*/*H*. For *d_p_*/*H* = 0.075, the range is rather large (*Re_c_*_1_ = 180 and *Re_c_*_2_ = 600), whereas for *d_p_*/*H* = 0.175, the range is rather small (*Re_c_*_1_ = 290 and *Re_c_*_2_ = 350). Larger particles, thus, rapidly transit from the face-centered migration regime to both the face-centered and corner migration regimes. These observations are also consistent with the results that we obtained in bi-disperse suspensions with a high particle to particle size ratio [24] where small particles did not focus toward the face-centered equilibrium positions already occupied by the large particles.

**Figure 5:**
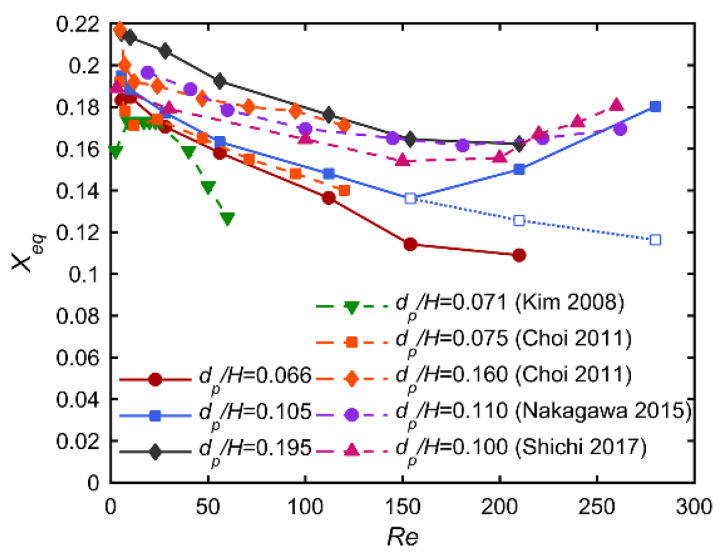
Particle equilibrium position *Xeq* (distance between the particle center and the nearest channel wall) for different particle to channel size ratios with respect to *Re*. Solid lines correspond to the face-centered equilibrium positions, while the dashed lines indicate the newly-arisen equilibrium positions near the channel corners.

Figure 5 presents the face-centered equilibrium position with respect to *Re* for different particle to channel size ratios. The equilibrium position is here defined as the distance between the center of a particle focalized at a face-centered equilibrium position and its nearest channel wall. Results from Kim et al. [25], Choi et al. [26], Nakagawa et al. [17], and Shichi et al. [16] are also shown in this figure for comparison.

It can be seen that, as *Re* increases, the equilibrium position moves outward to the channel wall first (*Re* < 100) and then inward to the channel centerline for higher *Re* (*Re* > 200). This evolution is in good agreement with previous numerical results of Nakagawa [17] and experimental ones of Shichi [16], obtained with particles and channels ten times greater and also with previous experimental analyses of Kim [25] and Choi [26] conducted at lower *Re* ranges, respectively, below 60 and below 120. Pan et al. [18] and Yuan et al. [19] proposed a theoretical analysis to understand the outward shift of the face-centered equilibrium positions. Two lift forces are dominant in the first stage of migration: the shear-induced lift force that pushes the particle toward the channel wall, and the wall-induced lift force that prevents the particle from touching the wall. The balance of these two forces determines the equilibrium position. Since the shear-induced lift force increases more than the wall-induced lift force when increasing *Re*, the face-centered equilibrium positions, thus, move toward the wall. To understand the inward shift at higher *Re*, Pan et al. [18] and Yuan et al. [19] analyzed experimentally and numerically the confined flow induced by a focused particle in a microchannel. They highlighted that the streamline structure in the vicinity of the particle could be very different from Poiseuille flow at higher *Re*. At low *Re* (~21), a vortex-like flow and a reverse flow are present upstream and downstream from the particle, respectively. As *Re* increases, a small vortex is formed near the particle on the downstream side (at *Re* = 63), growing gradually and finally replacing the reverse flow regime with a vortex flow regime (*Re* > 105). Meanwhile, the upstream vortex becomes stronger and occupies a greater volume with increasing *Re*. At low *Re* (<100), Poiseuille flow is dominant since the particle-induced secondary flow is relatively weak. However, for higher *Re* (>105), the particle-induced upstream vortex is strong enough to push the particle away from the wall. As a result, the distance between the focused particle and the channel wall increases once *Re* exceeds a critical value, which is in the range of 100–200 and depends on the particle to channel size ratio. According to Yuan et al. [19], the secondary flow induced by the larger particle is much stronger and occupies larger space in the channel when compared with that of a smaller particle, producing a stronger hydrodynamic resistance force on the particle and inducing a lower critical *Re_c2_*.

### 3.3. Effect of Particle Volume Fraction on the Lateral Migration Process

**Figure 6:**
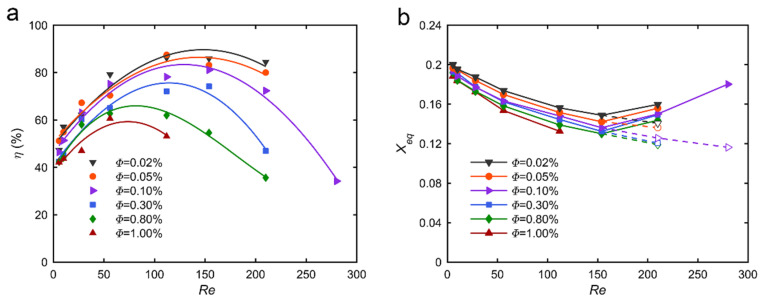
(**a**) Focusing degree and (**b**) equilibrium position with respect to *Re* at *d_p_*/*H* = 0.11, *z*/*H* = 1000, and different particle volume fractions. Solid lines correspond to the face-centered equilibrium positions, while the dashed lines indicate the newly-arisen equilibrium positions near the channel corners.

Figure 6 presents the focusing degree at *z*/*H* = 1 000 (a) and the equilibrium position (b) with respect to *Re* for a particle to channel size ratio *d_p_*/*H* = 0.11 and for six different particle volume fractions ranging from 0.02% to 1%.

The different focusing degree curves are very similar. The focusing degree first increases with increasing *Re*, reaches a maximum at a given critical Reynolds number *Re_c_*_1_, and then decreases due to a switch in the migration regime and, thus, the arising of other equilibrium positions. However, increasing the particle volume fraction decreases the maximum focusing degree *η* and decreases the critical Reynolds number *Re_c_*_1_: *Re_c_*_1_ ~ 150 and *η* ~ 90% at *Φ* = 0.02% whereas *Re_c_*_1_ ~ 56 and *η* ~ 60% at *Φ* = 1%. This phenomenon may be explained by the “space constraint” effect. For a given length of channel, only a certain number of particles can occupy the four face-centered equilibrium positions with a corresponding interparticle spacing in the streamwise direction regulated by interparticle forces. Further increase of the particle volume fraction (and, thus, of the particle number in the given volume of the channel) leads to stronger particle-particle interactions, which prevent the extra particles to reach the face-centered equilibrium positions. These particles, thus, stay, after the first stage of migration, in regions where the net inertial lift force is smaller, such as the unstable equilibrium positions or the annulus along the perimeter of the channel. This can be observed on the particle distributions corresponding to higher particle volume fractions for which the particles laterally migrate and concentrate on an annulus close to the channel perimeter, as is the case at lower particle volume fractions. However, their cross-lateral migration inside the annulus toward the face centers is limited.

As shown in Figure 6b, the different equilibrium position curves are very similar as well. However, the face-centered equilibrium positions are shifted toward the channel walls with an increasing particle volume fraction. The underlying mechanism of this phenomenon, which supposes a decrease of the wall-induced lift force or an increase of the shear-induced lift force in this range of *Re*, is still unclear. A dependence of these lift forces to the particle volume fraction has not yet been mentioned in the literature.

### 3.4. Longitudinal Ordering Process

It is well known that inertial migration at moderate Reynolds numbers simultaneously drives lateral migration toward four equilibrium positions located near the centers of the channel faces and longitudinal ordering of particles [18,22]. According to Gao et al. [23], a different regime of migration occurs at small Reynolds numbers (*Re* < 5) in square microchannels where particles focus on the channel centerline. No trains have been observed during this regime of migration. As seen in the previous section, a third and even a fourth migration regime have been highlighted at high Reynolds numbers. The longitudinal ordering of spherical particles when *Re* varied from 10 to 280 is, thus, analyzed in this section.

**Figure 7:**
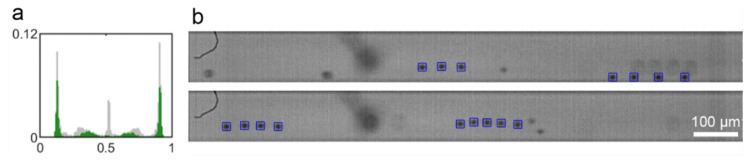
(**a**) Probability density functions of all the 8.44-µm particles in grey and solely the particles in trains in green. (**b**) Channel pictures of 8.44-µm particles, in the channel midplane for *Re* = 280 at a distance from the channel inlet *z*/*H* = 1000 and a particle volume fraction *Φ* = 0.05%.

**Figure 8:**
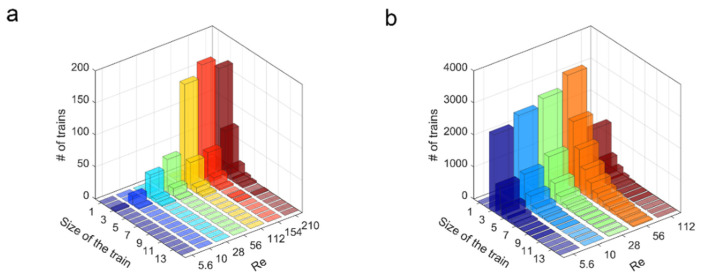
Composition of the trains with respect to *Re* observed at *z*/*H* = 1000 for *d_p_*/*H* = 0.11 with (**a**) *Φ* = 0.05% and (**b**) *Φ* = 1%, respectively.

Trains are identified when three or more particles are aligned with a regular interparticle spacing. In our previous work [23], it was observed that the fraction of particles in trains first increases with increasing Reynolds number, reaches a maximum and decreases for higher Reynolds numbers. This evolution was shown to be similar to that of the corresponding focusing degree. We even highlighted in Reference [23] that the Reynolds numbers at which the focusing degree and the fraction of particles in trains found their maximum are identical. This means that, at Reynolds numbers higher than the critical Reynolds number (*Re_c_*_1_ ~ 120 in the present conditions), when particles migrate toward either the four face-centered equilibrium positions or the eight positions located on the heteroclinic orbit, the fraction of particles in trains decreases. To better understand the link between lateral migration and train formation, train positions were extracted from the particle distributions. Figure 7 shows the probability density functions of all the 8.44-µm particles in grey and solely the particles in trains in green in the mid-plane of the channel at *Re* = 280. Trains are observed both on the four face-centered equilibrium positions (green peaks near the walls) and on the intermediate equilibrium positions (green hills near the center). This observation was confirmed by the pictures in Figure 8b where trains were identified on both types of equilibrium positions.

Particle longitudinal ordering is, thus, reduced at Reynolds number higher than *Re_c_*_1_ but, nevertheless, still exists. The reduction of the number of trains above *Re_c_*_1_ is certainly linked to the higher number of equilibrium positions. Since particles are distributed on more equilibrium positions, they are less concentrated on each equilibrium streamline and are, thus, less likely to interact with each other to form trains.

Figure 8 presents the composition of the trains for different Reynolds numbers ranging from 5 to 210.

Regardless of the Reynolds number and the particle volume fraction, the vast majority of the trains observed in a given distribution was formed by three particles. Trains of four particles are less likely encountered. Trains composed of more than five particles are rare. A similar phenomenon was observed by Matas et al. [20] in a circular channel, where the percentage of particles in trains showed an overall decreasing tendency with the size of the train. Depending on the particle to channel size ratio, the most frequently encountered trains, according to Matas, are composed of five (*d_p_*/*D* = 1/17) or three particles (*d_p_*/*D* = 1/19). This observation is also consistent with the numerical results of Gupta et al. [27] who highlighted that the maximum number of stable aligned particles per train is equal to three in similar confined conditions. The percentage of three-particle trains, even if these trains were still in the majority, was decreased at higher particle volume fractions, where longer trains were more frequently encountered. Finally, the most probable length of the trains seems to be similar for all the studied Reynolds numbers, indicating that its dependence on *Re* is weak.

## 4. Conclusions

In this work, we have conducted a systematic investigation on the inertial behavior of spherical particles of different sizes (5.3, 8.44, and 15 µm) at *Re* ranging from 5 to 280 in an 80-µm sized micro-channel. 

First, it has been confirmed that, at a fixed distance from the channel inlet, a specific Reynolds number exists, at which the focalization is optimal, i.e., at which the ratio of particles located on the four equilibrium positions is the highest. Under this value, the migration is not fully-developed and, over this value, new additional equilibrium positions near the channel corners arise. Smaller particles were demonstrated to be more prone to locate at these unstable equilibrium positions than larger ones. It has been shown that this “optimal” Reynolds number depends on the particle to channel size ratio and on the particle volume fraction. Higher particle concentration can lead to a lower focusing degree and to the shift of the equilibrium positions toward walls.

Second, particle ordering phenomenon was observed not only on the face-centered equilibrium positions, but also on the equilibrium position near the corners. Longer trains were more frequent at higher particle volume fractions.

Our investigations at high *Re* have brought new insights on the inertial behavior of particles in suspension in square microchannels, which will be helpful for an effective design of high-throughput microsystems for particle concentration or separation in lab-on-a-chip applications.

## Data Availability

The data presented in this study are available on request from the corresponding author.
